# Supplementary figures and images for: RIO-kinase 2 is essential for hematopoiesis
Source: PLoS One. 2024 Apr 2;19(4):e0300623. doi: 10.1371/journal.pone.0300623 (PMC10986946; doi:10.1371/journal.pone.0300623)

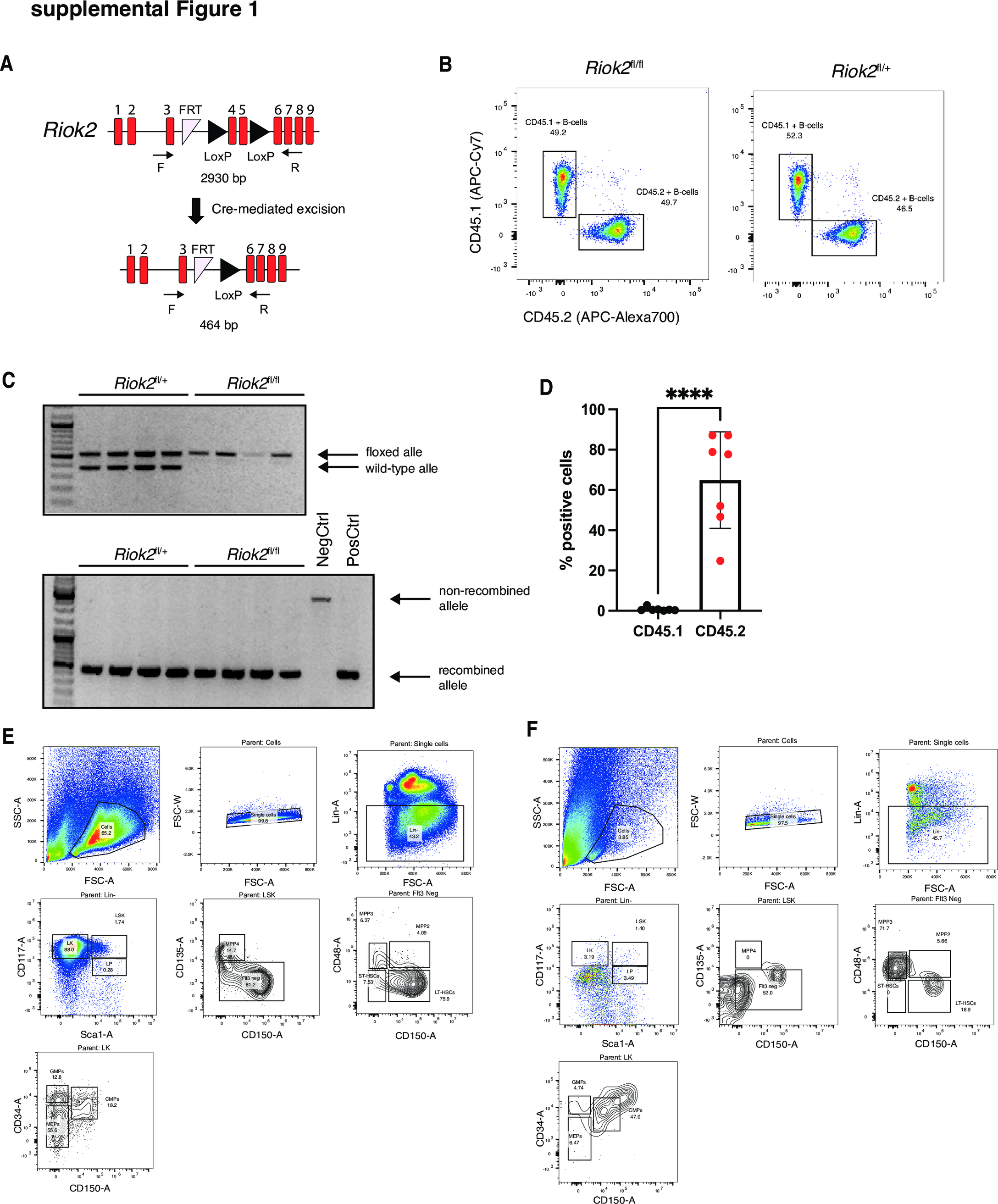

Supplement: S1 Fig — A) Schematic overview of the Riok2fl/fl locus. Exons 4 and 5 are flanked by LoxP site that are excised after the induction of Cre recombinase. B) Representative FACS plots showing the engraftment of Riok2fl/fl and Riok2fl/+ B-cells 4 weeks after transplantation using CD45.1/CD45.2 staining. C) Agarose gel showing the genotyping of the transplanted bone marrow of ROSA26::CreERT2, Riok2fl/+ and Riok2fl/fl mice used in the competitive bone marrow transplantation assay after termination of the experiment. Arrows indicate wild-type and floxed as well as recombined and non-recombined alleles. The lanes between the bottom and top gels are matched and represent the same mice. D) Bar graph depicting the percentage of CD45.1 or CD45.2 positive cells in lethally irradiated mice transplanted with Riok2fl/fl; Rosa26::CreERT2. Data is represented as mean ± standard deviation (SD) (n = 8). ****: p<0.0001 by Unpaired t-test. E) Gating strategy for HSPC population identification and representative result for mice injected with corn oil. F) Gating strategy for HSPC population identification and representative result for mice injected with tamoxifen and experiencing symptoms of bone marrow failure. (TIF) [file pone.0300623.s001.tif]

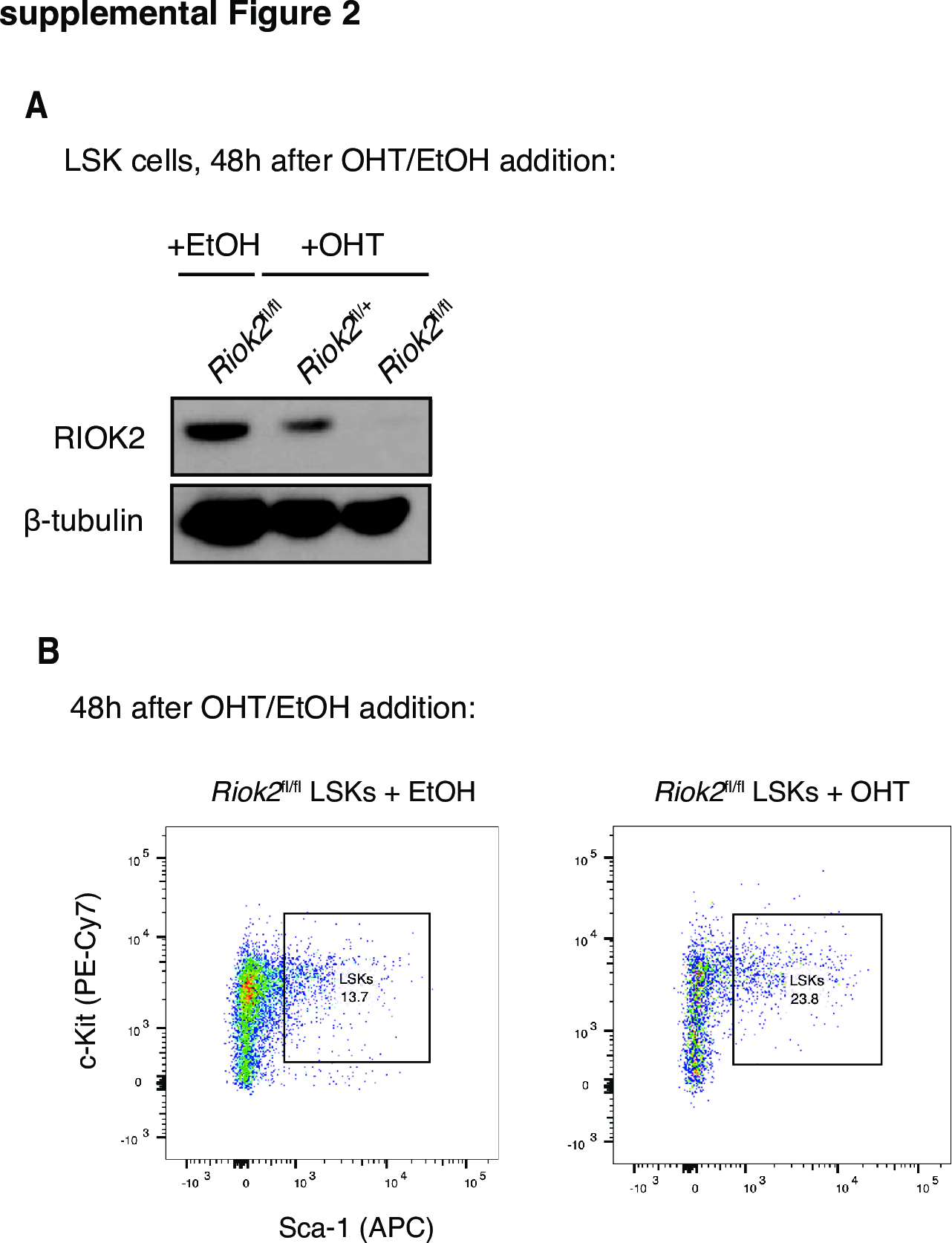

Supplement: S2 Fig — A) Immunoblot showing RIOK2 protein levels in LSK cells cultured in X-VIVO medium from the indicated genotypes 48 hours after the addition of either EtOH or OHT to the culture medium. B) Representative FACS plots for X-VIVO cultured LSK cells 48 hours after the addition of EtOH or OHT to the culture medium. (TIF) [file pone.0300623.s002.tif]

Supplemental Figure 1C

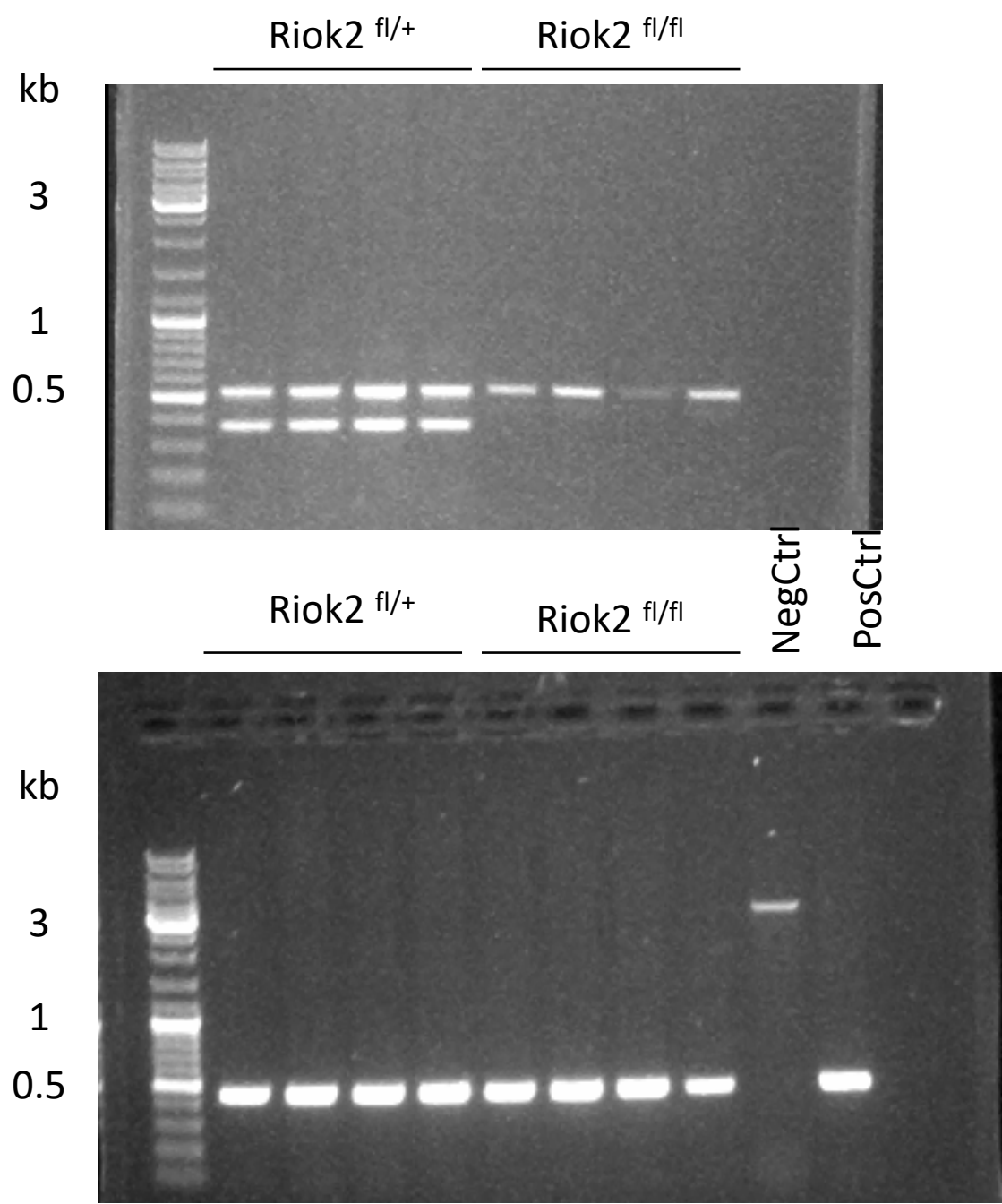

Supplemental Figure 2A

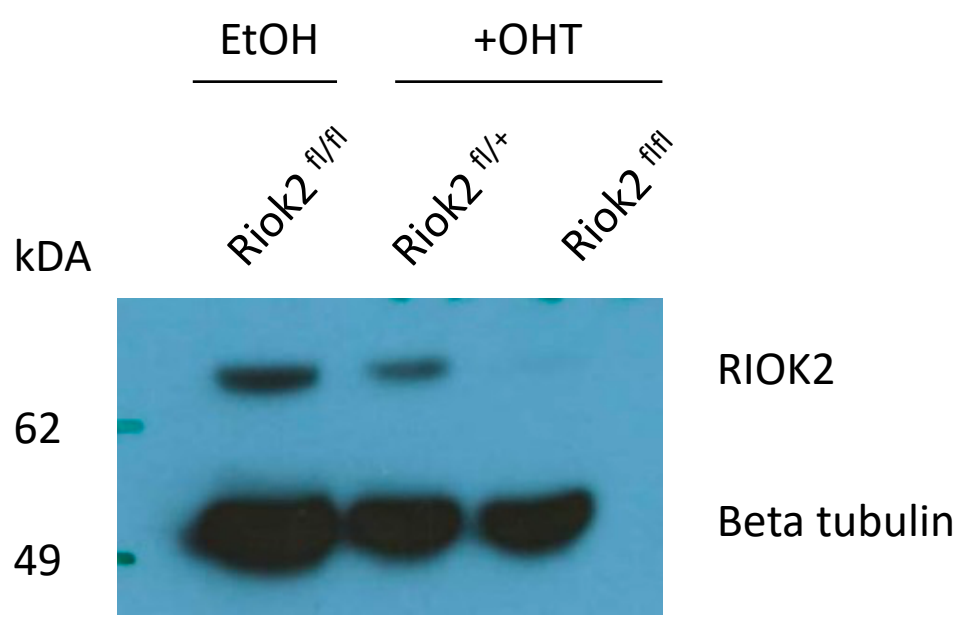

Supplement: S1 Raw images — (PDF) [file pone.0300623.s003.pdf]
