# Supplementary material for: RIO-kinase 2 is essential for hematopoiesis
Source: PLoS One. 2024 Apr 2;19(4):e0300623. doi: 10.1371/journal.pone.0300623 (PMC10986946; doi:10.1371/journal.pone.0300623)
Supplement: Attachment — Submitted filename: Response to Reviewers.docx [file pone.0300623.s005.docx]

**Response to Reviewers**

**Reviewer #1:**

**In Figure 1, we propose that both Riok2fl/fl; Rosa26::CreERT2 and Riok2fl/+; Rosa26::CreERT2 have the effect of downregulating RIOK2 gene expression. Why was Riok2+/+; Rosa26::CreERT2 mice not used as a control?**

Heterozygous deletion of *Riok2* does not influence mature hematopoietic cells in our competitive transplants. Figure 1B shows that the CD45.1:CD45.2 ratio is stable across all timepoints and for all cell types in the Riok2^fl/+^ competitive experiments. Additionally, as shown in Figure 1 D and E, we do not observe differences in mouse survival and bone marrow composition upon loss of one allele of *Riok2* compared to wild-type mice. Therefore, we used *Riok2fl/+* as a control for the effect of deleting both alleles of *Riok2*.

**In Supplementary Figure 1, should there be additional images demonstrating the efficiency of RIOK2 gene knockdown in Riok2fl/fl; Rosa26::CreERT2 and Riok2fl/+; Rosa26::CreERT2 mice, rather than solely relying on DNA-level identification?**

We agree with the reviewer that it would be nice to show. However, unfortunately, it is not possible to obtain enough cells to perform immunoblotting of RIOK2 on material from the competitive transplant, because the RIOK2 knockout cells were rapidly outcompeted by the non-recombined wild-type cells. Importantly, we have shown in supplemental Figure 2A that tamoxifen treatment leads to a rapid and efficient depletion of RIOK2 protein on hematopoietic stem- and progenitor cells, supporting the efficiency of the deletion of both alleles of *Riok2.*

**In Figure 2, the experimental data regarding the impact of Riok2 knockout on HSC proliferation and differentiation is insufficient to support the authors' conclusions. Are there additional evidence available to substantiate these claims?**

We agree with the author that the claims were insufficient and have removed the model that concluded independent mechanisms and a differentiation block for hematopoietic stem- and progenitor cells and mature cells.

**Reviewer #2:** 

**The figures included in the main text are of such low quality that it is difficult to discern the text within the images. Enhancement of image clarity is necessary.**

We are sorry for this. The link provided in the downloaded pdf of the manuscript should have provided a high-resolution TIFF file of each of the figures. We have ensured that the figures in the revised version agree with the requirement of the journal.

Minor Points:
**1. In Fig S1C, concerning the lower image of genotyping, it is unclear what the genotype is for the control. Why do two lanes of ctrl display mismatched bands?**

The control bands are a negative (wild-type) control, which is the non-recombined locus as well as a positive (*Riok2fl/fl*) control. We have added additional description to the supplemental Figure 1C and its figure legend to enhance clarity.

**2. Is it critical to establish the baseline levels of CD45.1 and CD45.2 in mice before the transplantation process? Additionally, would it enhance clarity to more distinctly show the reduction of CD45+ cells following radiation?**

We have used appropriate controls to correctly set up the FACS gates for determination of CD45.1 and CD45.2 levels. In competitive transplantation it is not common practise to establish baseline levels. The assay is to establish the competition and not the total levels of each population.

**3. Some of the statistical charts, like those in Figure 1B, 1C, and FigS1D, would benefit from an analysis of significance to support the findings.**

We have performed additional statistical analyses for the figures requested by the reviewer and added appropriate descriptions to the figures and figure legends.
